# Bobolink (*Dolichonyx oryzivorus*) Declines Follow Bison (*Bison bison*) Reintroduction on Private Conservation Grasslands

**DOI:** 10.3390/ani11092661

**Published:** 2021-09-10

**Authors:** Rachel H. Kaplan, Kristen M. Rosamond, Sandra Goded, Alaaeldin Soultan, Alex Glass, Daniel H. Kim, Nico Arcilla

**Affiliations:** 1 International Bird Conservation Partnership, Monterey, CA 93940, USA; rachelhkaplan@gmail.com (R.H.K.); kristenrosamond@gmail.com (K.M.R.); sandra.goded@birdpartners.org (S.G.); 2 Crane Trust, Wood River, NE 68883, USA; alexander.glass@siu.edu (A.G.); texraptor@hotmail.com (D.H.K.); 3 Department of Biology, University of Missouri-St. Louis, St. Louis, MO 63121, USA; 4 Department of Ecology, Swedish University of Agricultural Sciences, 750 07 Uppsala, Sweden; alaaeldeen80@gmail.com; 5 Cooperative Wildlife Research Laboratory, Southern Illinois University, Carbondale, IL 62901, USA; 6 U.S. Fish and Wildlife Service, Pierre, SD 57501, USA; 7 Center for Great Plains Studies, University of Nebraska-Lincoln, Lincoln, NE 68588, USA

**Keywords:** Bobolink (*Dolichonyx oryzivorus*), conservation, American bison (*Bison bison*), domestic cattle (*Bos taurus*), reintroduction, Monitoring Avian Productivity and Survivorship (MAPS), grazing, grasslands, climate change

## Abstract

**Simple Summary:**

North American grassland birds evolved with American bison (*Bison bison*), until overhunting drove bison to near-extinction > 150 years ago. Bison have now been reintroduced to many areas that provide important nesting habitat for grassland birds, which are now among the most rapidly declining birds in North America. However, little is known about bison interactions with birds such as Bobolinks (*Dolichonyx oryzivorus*), obligate grassland nesting songbirds of conservation concern. Using data collected over an 18-year period, we assessed the effects of bison reintroduction, together with other land management and climate factors, on Bobolinks in a private conservation area comprising 24 km^2^ of native grasslands in the North American Great Plains. In grasslands where bison were reintroduced, Bobolink abundance (adult numbers) declined by 62%, and productivity (juvenile numbers) declined by 84%. By contrast, Bobolink populations remained stable over the same time period in adjacent grasslands where bison were not reintroduced. Bobolink abundance and productivity increased in years following warmer and wetter winters, but nevertheless declined over time in grasslands where the bison population doubled. Where bison are reintroduced and confined in high densities, overgrazing, trampling, and related impacts may drive severe declines in Bobolinks and other grassland birds of conservation concern.

**Abstract:**

Among the most rapidly declining birds in continental North America, grassland birds evolved with American bison (*Bison bison*) until bison nearly became extinct due to overhunting. Bison populations have subsequently rebounded due to reintroductions on conservation lands, but the impacts of bison on grassland nesting birds remain largely unknown. We investigated how bison reintroduction, together with other land management and climate factors, affected breeding populations of a grassland bird species of conservation concern, the Bobolink (*Dolichonyx oryzivorus*). We quantified population changes in Bobolinks over an 18-year period in conservation grasslands where bison were reintroduced, compared with adjacent grasslands grazed by cattle and where hay was harvested after the bird breeding season. Four years after bison reintroduction, the bison population in the study area had doubled, while Bobolink abundance declined 62% and productivity declined 84%. Our findings suggest that bison reintroduction as a conservation strategy may be counterproductive in grassland fragments where overgrazing, trampling, and other negative impacts drive declines in grassland breeding birds. Where bird conservation is an objective, small grassland reserves may therefore be inappropriate sites for bison reintroduction. To maximize conservation benefits to birds, land managers should prioritize protecting grassland birds from disturbance during the bird breeding season.

## 1. Introduction

Grassland and farmland bird populations worldwide have declined steeply for decades [1,2,3], warranting urgent conservation action. In North America, grassland birds evolved alongside American bison (*Bison bison*; hereafter, bison), until overhunting drove bison to near extinction in the late 19th century. Reintroductions of species that have been extirpated as a result of human activities constitute a powerful conservation tool. For example, reintroduction efforts have enabled wolves (*Canis lupus*) to repopulate a national park in the United States [4] and African wild dogs (*Lycaon pictus*) to repopulate a national park in Mozambique [5]. Likewise, the reintroduction of bison to many areas in North America is celebrated as a conservation success story, as bison numbers have rebounded from fewer than one thousand to hundreds of thousands of individuals today [6]. However, the vast majority (>95%) of bison today exist in commercial herds used for meat production, while <5% live on conservation lands constituting <1% of their historical range [7]. Reintroduction efforts highlight the ecological importance of bison and evidence that popular interest in bison encourages public support for conservation [8,9]. However, grassland bird responses to bison grazing have rarely been studied due to the absence of bison from most North American grasslands for over a century [10].

Historically, tens of millions of migratory bison roamed the North American Great Plains, functioning as keystone herbivores in native grasslands [7,11]. Reintroductions of keystone species, which have significant impacts on ecosystem function, organization, and stability, have rarely been studied in terms of their ecosystem-level effects [12]. During European settlement of the Great Plains in the 1800s, domestic cattle (*Bos taurus*; hereafter, cattle) replaced bison as the dominant grazing species, but there are ongoing efforts to reintroduce bison to additional portions of their former range [6,7,13]. However, most bison today are managed as livestock rather than wildlife, and their confinement within fenced pastures and protection from predation has raised questions about their ecological roles and interactions with other species in areas where they have been reintroduced [14,15]. Investigating the responses of grassland birds to bison reintroduction thus provides an important opportunity to understand the effects of a long-absent keystone herbivore in modern land management systems on other grassland species of urgent conservation concern. This is particularly pressing given that >70% of grasslands worldwide, and >80% of grasslands in North America, have been converted to agriculture [16,17], resulting in the status of temperate grasslands as among the most endangered and least protected ecosystems on earth [18,19,20].

Given the relative lack of protection for grasslands, most of which are privately owned [21,22], reintroducing bison may bring more attention and support for grassland conservation, benefiting grassland birds. Public interest and funding for popular species have been leveraged to meet multiple conservation objectives in other cases, such as Northern Bobwhites (*Colinus virginianus*) acting as an umbrella species for some other species of grassland and shrubland birds [23]. However, management focused on one species may not benefit other species in the same ecosystem [24]. For example, management for ducks in grasslands may not benefit grassland songbirds [25]. Furthermore, management for one species may also have adverse impacts on others, such as the case in which forests managed for Red-cockaded Woodpeckers (*Picoides borealis*) led to the extirpation of Wood Thrush (*Hylocichla mustelina*) that had previously nested in these forests [26]. Likewise, understanding the effects of management for bison on vulnerable grassland bird species is important for informing bird conservation efforts.

Grasslands are dynamic ecosystems in which both land management (e.g., grazing, hay harvest, patch burning [27]) and climate factors (e.g., temperature, precipitation, drought [28]) may be important predictors of songbird population dynamics [29]. As top herbivores, bison and cattle both have important modifying effects on birds and other wildlife [30]. Research to date indicates that bison tend to be more mobile and less dependent on water sources than cattle, and that bison graze on different plant communities, potentially disseminating native plants more widely across the landscape [31]. There are moreover profound differences between management practices for cattle and bison, respectively [30,32]. Cattle domestication began ~10,000 years ago [33] and their confinement often requires minimal fencing. Most bison are now considered semi-domesticated, but are by contrast much more difficult to move and contain, and their confinement requires high, electrified fencing, which is costly to install. Cattle management typically includes rotational grazing, where cattle occupy grasslands for a period of months before their removal, after which plant and animal communities recover. By contrast, bison management often includes extended or continuous grazing by breeding herds that may occupy grasslands year-round. In addition, because managed bison survive and reproduce at high rates, managers in limited grassland areas must frequently remove bison to prevent overpopulation and habitat degradation due to overgrazing and trampling [32,33,34,35].

While habitat management for bison may have significant impacts, little is known about the effects of bison management on grassland ecosystems and species in practice [32]. However, several previous studies have examined effects of reintroduced bison on birds in grasslands that bison formerly inhabited but from which they have been absent for >150 years. A 1-year study of bison reintroduction effects on grassland bird density and habitat use, mammal habitat use, and plant composition and structure found few short-term ecological effects of bison but a significant increase in visitor interest [9]. A 4-year study of grassland bird nests found that nest density and success decreased 2 years after bison reintroduction [36]. A 23-year study of bird abundance in grasslands where bison were reintroduced found Upland Sandpiper (*Bartramia longicauda*) and Grasshopper Sparrow (*Ammodramus savannarum*) increases but Dickcissel (*Spiza americana*) declines and the near extirpation of Henslow’s Sparrow (*Ammodramus henslowii*) [11]. To date, however, no previous published studies have measured the effects of bison reintroduction on both the abundance and productivity of grassland birds. Bird abundance (annual numbers of unique adult individuals) and productivity (annual numbers of unique juvenile, or hatch-year, birds produced by breeding adults), are important avian vital rates [37]. Measuring such demographic parameters in response to external factors enables an understanding of bird population dynamics that in turn provides important information for conservation and adaptive management.

Here, we examine the effects of bison reintroduction on the abundance and productivity of the Bobolink (*Dolichonyx oryzivorus*), a neotropical migratory songbird and obligate grassland nesting species of conservation concern [38]. North American Breeding Bird Survey (BBS) data indicate that Bobolink populations have declined globally at an average rate of 2% per year across their breeding range [39], culminating in a decline of ~60% over the past ~50 years [40]. Bobolink conservation priorities include identifying and fostering conditions on the breeding grounds that improve their productivity [37]. Primary factors driving Bobolink declines include the conversion of grasslands to row agriculture and associated habitat loss and degradation on their breeding grounds, where they often build nests at the base of large flowering plants [38,41,42]. Although Bobolinks disperse from heavily grazed grasslands [43,44], past research has suggested that light grazing by bison may be compatible with Bobolink conservation [45]. We took advantage of a unique opportunity to quantify changes in Bobolink abundance and productivity over an 18-year time period (2002–2019) during which bison were reintroduced (in 2015) to a global priority Important Bird Area [46] in Nebraska, in the North American Great Plains. 

We hypothesized that reintroducing bison as the keystone native herbivore would improve the conservation value of grassland as habitat for Bobolinks and other declining grassland bird species. We predicted that if grazing bison improved the conservation value of grasslands for birds, breeding birds would respond by exhibiting corresponding increases in abundance and/or productivity. We tested our hypothesis by first quantifying and comparing Bobolink abundance and productivity both before and after the reintroduction of bison to our study area. Second, because Bobolinks are declining globally [38], we checked Bobolink population changes over time in our study area against state-wide (e.g., Nebraska BBS) Bobolink population data during the time period of our study, to allow us to distinguish any local population changes from any background decline. Third, we compared Bobolink abundance and productivity in grasslands grazed by bison with adjacent grasslands grazed by cattle and grasslands where hay was harvested after the bird breeding season. Finally, we investigated whether additional management and climate parameters influenced Bobolink abundance and/or productivity in our study area. 

## 2. Materials and Methods

### 2.1. Study Area

**Figure 1:**
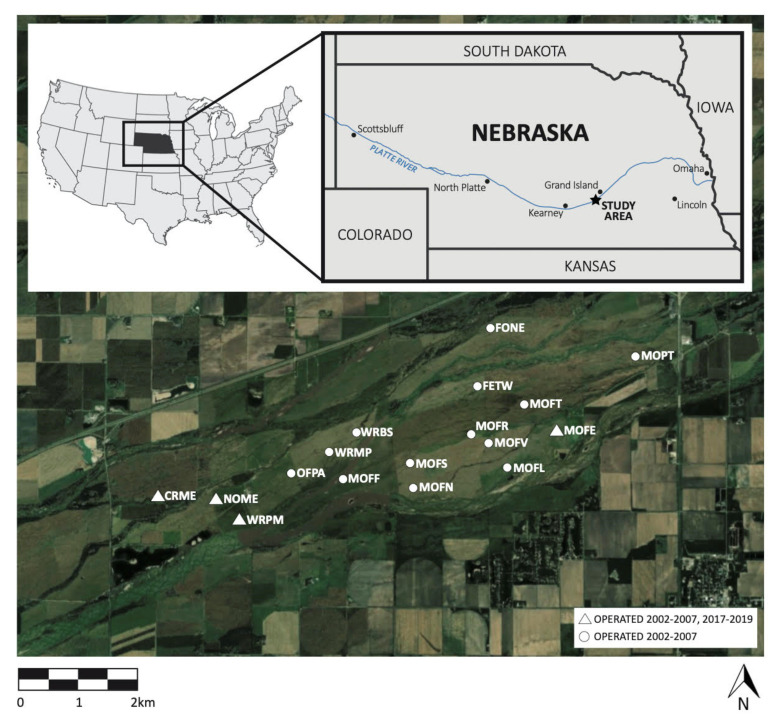
Map of the study area showing bird sampling sites in the Platte River Valley, Nebraska, USA. Circles represent ~8 ha sites run 2002–2007; triangles represent ~8 ha sites operated in both 2002–2007 and 2017–2019. Map produced by R.H.K. using ArcGIS software release 10.6, Redlands, CA: Environmental Systems Research Institute.

We conducted research within a 2430 ha fragment of riparian tallgrass prairie in the Platte River Valley, Nebraska, USA (Figure 1), an Important Bird Area and remnant of the critically endangered North American Central and Southern Mixed Grasslands ecoregion. While much of the surrounding land has been converted to row agriculture, especially corn (*Zea mays*), these grasslands were protected in 1978 as a private conservation area managed by the Crane Trust, a nonprofit organization with the mission of protecting and maintaining habitat for Whooping Cranes (*Grus americanus*), Sandhill Cranes (*Antigone canadensis*), and other migratory birds. Ephemeral disturbances such as grazing by migratory bison, seasonal flooding, and wildfires [47,48] historically structured these grasslands, where managed grazing, haying, and patch burning now control woody encroachment and maintain grasslands. Cattle were the sole grazers until April 2015, when the Crane Trust reintroduced a herd of 53 bison in a 140 ha enclosure [13]. By April 2019, the bison herd size had more than doubled to 110 bison [49] in an area of 336 ha [50]. Grazing by cattle and bison often overlapped with the songbird breeding season, whereas haying and burning typically took place outside the bird breeding season, in the fall and spring, respectively. 

### 2.2. Bird Sampling

Between 2002 and 2019, we collected data on birds via constant-effort mist-netting, using protocols standardized by the Monitoring Avian Productivity and Survivorship (MAPS) program to assess avian population parameters and vital rates [51,52]. We identified captured birds to species and fitted them with a uniquely numbered aluminum band (ring) issued by the U.S. Geological Survey Bird Banding Laboratory under U.S. federal bird banding permit 23224. We recorded birds’ demographic information and biometric measurements and then released birds at the site of capture [53]. We deployed either 12 (2002–2007) or 10 (2017–2019) mist nets (12 m × 3 m, 4-tier, 30 mm mesh) within an ∼8 ha area at each site. We operated nets approximately once every 10 days for a capture period of 6 hours beginning at local sunrise (~06:00 h). Effort was consistent for each sampling event, except when nets were occasionally closed during periods of high winds, heavy precipitation, or lightning storms, in which case we compensated by adding equal effort to a subsequent sampling occasion. We sampled birds at 17 sites during the bird breeding season within an 80-day period from late May to early August. Between 2002 and 2007, we sampled 3 sites for 6 consecutive years and 14 sites for periods ranging between 1 and 5 years. From 2017 to 2019, we replicated bird sampling at 4 sites with different management treatments (bison grazing, cattle grazing, haying) for 3 consecutive years.

### 2.3. Analytical Approach 

Capture-mark-recapture (CMR) sampling is a powerful tool for estimating animal abundance. Novel approaches to capture-recapture analyses are increasingly used to estimate abundance of open populations of animals [54,55,56,57,58], expanding earlier uses of capture-recapture analyses that focused on estimating survival [54,59]. Habitat types, seasons, and species’ behavior and life histories may influence mist net capture rates [37,60,61]. Bearing in mind these caveats, appropriate use of mist net CMR data allows us to standardize quantitative data on bird populations, including information on birds’ sex, age, and biometrics, enabling the analysis of demographic parameters to generate insight into ecological processes. 

We used CMR data to compare Bobolink abundance and productivity before bison reintroduction (using data from 2002 to 2007) with that in grassland where bison were reintroduced in 2015 (using data from 2017 to 2019). We then checked our CMR data against Nebraska BBS data collected during the same time period in order to distinguish any background level of population change in Nebraska from any population changes we identified locally at our study sites. Next, we compared Bobolink abundance and productivity in bison-grazed grasslands with that in grasslands grazed by cattle and grasslands that were hayed after the bird breeding season. Finally, we investigated the effects of specific land management (grazing, burning, haying) and climate (temperature, precipitation, drought) parameters using Bobolink adult abundance (hereafter, abundance) and juvenile abundance (hereafter, productivity) as response variables [37,62,63,64].

### 2.4. Statistical Analyses

We used Bobolink CMR data across all 17 sites, removing recaptures of individuals that occurred at the same site during the same year to avoid double-counting. We corrected for annual capture effort by dividing the number of captures by the cumulative mist net-meter-hours for the given year [63,64]. We divided the banding season into two halves for data analysis, in which the first half (21 May–29 June) comprised adults arriving and establishing breeding territories and nests, and the second half (30 June–8 August) comprised both adults and juvenile birds that had fledged from successful nests, e.g., [37,63,64]. We conducted all statistical analyses in R [65]. 

We used a t-test to determine whether Bobolink populations changed after the reintroduction of bison, whether local population changes differed from statewide population changes, and whether Bobolink abundance in grasslands grazed by bison differed from those in grasslands grazed by cattle or grasslands where hay was harvested after the bird breeding season. We considered *p*-values < 0.05 sufficient evidence to reject the null hypothesis of no relationship between predictor and response. Subsequently, we tested whether changes in Bobolink abundance and productivity were associated with land management and/or climate factors using generalized linear mixed models (GLMMs). We tested for correlations between land management and climate parameters with a Spearman’s correlation test to account for the non-normal distribution of parameters, using the package psych [66]. We removed correlated variables and used the uncorrelated variables (*r*_s_ < |0.7|) to perform GLMMs. Several attempts have been made to set a threshold value for correlation in ecological systems, yet there is still no agreement on a single correlation value [67,68]. Some studies recommend *r*_s_ > 0.6, and others *r*_s_ > 0.8 [69]. However, there is general agreement that *r*_s_ > 0.7 is an appropriate value for assessing correlation between predictors [70] and including variables that have ecological importance, and we thus used this value in our study. 

GLMMs are useful for fitting ecological data, as they account for random effects and handle data that is not normally distributed through a flexible approach that overcomes problems such as observational dependency and heteroscedastic variance by including sites and years as random factors [71,72]. For some variables (e.g., months since grazing), we expected a non-linear relationship with abundance, and we also expected interactions between other variables (e.g., months since grazing and months since burning). Therefore, we fitted GLMMs with all possible combinations including linear, quadratic, and interaction terms. We standardized all of the continuous variables prior to analysis for better convergence using the “scale” function, wherein predictor units of measurement were removed to allow for comparison of coefficients regardless of their actual scales [73]. 

After analyzing different possible combinations including linear, quadratic, and interaction terms, we used Akaike’s Information Criterion (AIC) [74] to select the models that best fit the data. For analysis of adults, we used a negative binomial distribution to account for overdispersion of the data using the package glmmTMB [75]. We also used the package glmmTMB to run logistic models for productivity (i.e., juvenile Bobolinks) after we converted these data into presence/absence. We created a binomial index to quantify productivity, assigning juvenile birds a value of “1” and adults a value of “0”. We ran all possible combinations, including both year and site as random factors, but models with year as a random factor failed to converge. Final models included both linear and quadratic terms, as well as site as a random factor to account for non-independent observations within each site. We reported the z-score for each predictor, which can be used as a surrogate for predictor importance on the model. We considered *p*-values < 0.05 as significant, as stated above. 

### 2.5. Land Management Parameters

We analyzed six habitat and land management parameters relevant to our study area (Table 1). Three variables represent time (months) since grazing, haying, or burning. We set the maximum time since disturbance value for grazed, hayed, and burned pastures at 180 months (15 years) because management actions that occurred earlier were unlikely to have a predictable effect on habitat structure [76]. In addition, we created a grazing intensity variable representing livestock (cattle or bison) stocking rate for grazed pastures in animal unit months per hectare (AUM/ha), where one AUM equals the forage requirement for one adult and calf pair for a 1-month period [77,78]. Grazing intensity is represented by AUM/ha at each study site at the time of data collection; a value of 0 represents sites that had no grazing during MAPS data sampling. We also created a categorical variable to distinguish remnant native grasslands with no major disturbance history from restored grasslands on land previously used for row agriculture or planted with exotic grasses. Additionally, we included a variable representing the percentage of trees within a 200 m radius of the center of each banding site, which we estimated by using historical aerial imagery for the time period of this study [79] and Habitat Structure Assessments (HSAs) conducted as part of the MAPS protocols [52]. 

### 2.6. Climate Parameters

We obtained climate data from the National Oceanic and Atmospheric Administration (NOAA) [80] online database for south-central Nebraska (station KGRI, 40.968° N, −98.340° W, Central Nebraska Regional Airport in Grand Island, Nebraska; approximately 21 km northeast of our research site). Using this database, we created eight climatic variables that maximized the amount of relevant climatic information while reducing high collinearity (Table 2); [81,82,83,84,85,86]. These variables included maximum temperature of warmest month (°C; July), minimum temperature of coldest month (°C; January), precipitation of wettest month (mm; May), and precipitation of driest month (mm; January).

We also used NOAA’s Palmer Drought Severity Index (PDSI) for Nebraska’s Climate Division 5 (Central) to further elucidate the effects of regional drought conditions in the nonbreeding and breeding seasons. The PDSI is a widely used measure of drought intensity which incorporates both local temperature and precipitation data into a single index, providing a more comprehensive metric of moisture conditions than either variable measured in isolation [87,88]. As PDSI for a given month represents cumulative drought conditions of the preceding months, our PDSI August variable was selected as a representation of breeding season (May–July) drought intensity [89]. For our PDSI nonbreeding variables, we averaged PDSI values for the nine months prior to the breeding season (August–April) [90] to create one variable for the current year and one for the previous year. Finally, in order to account for potential breeding season carryover effects, we calculated two variables representing average temperature and total precipitation of the previous breeding season, respectively [91]. Precipitation in the wettest month (May), as well as PDSI in spring and the breeding season, was strongly correlated with PDSI in the nonbreeding season (correlation coefficients (*r_s_*) > 0.70). As our August PDSI variable represented breeding season conditions, we selected PDSI in the nonbreeding season in order to gain a more complete picture of year-round drought conditions.

## 3. Results

Between 2002 and 2019, we captured a total of 885 individual Bobolinks (Table S1) including 730 adults, 148 juveniles, and 7 individuals whose age was not recorded. Of 717 adult Bobolinks of known sex, ~65% were males and ~35% were females (a ~2:1 male: female ratio). We recaptured a total of 58 (~13%) Bobolinks, ~81% of which occurred at the same site as that of initial capture. Juveniles (hatch-year birds) made up ~17% of total Bobolink captures, which was the equivalent of ~2 juveniles for every breeding female Bobolink. The proportion of new juveniles to total individual Bobolinks per year ranged from 0 in 2019 to ~28% (n = 63) in 2006 (mean = 16.6 ± 6.71 SE).

### 3.1. Bobolink Abundance and Productivity before and after Bison Reintroduction

**Figure 2:**
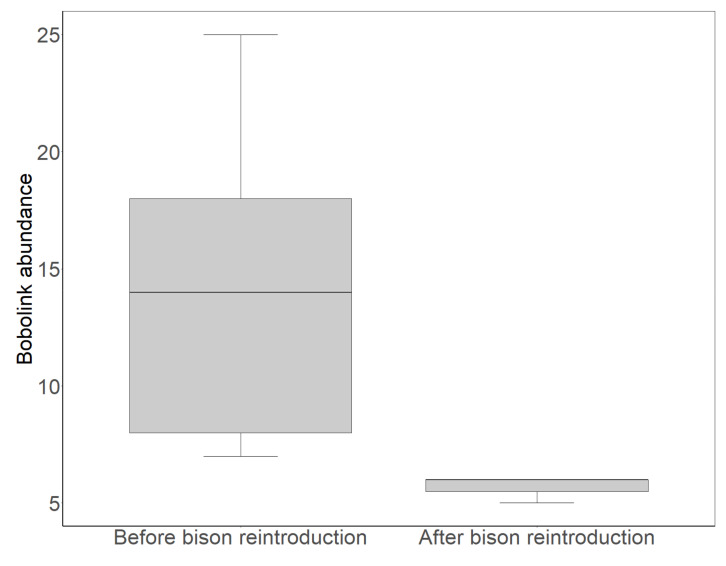
Boxplot showing Bobolink abundance (unique adult individuals captured/year) before (2002–2007) and after (2017–2019) bison reintroduction (2015) on conservation grasslands in Nebraska, USA. Black lines in boxes represent median values of abundance, while the lower and upper box edges represent the 25th and 75th percentile of the abundance, respectively. Error bars represent the 5th and 95th percentiles of the abundance.

Four years after bison reintroduction in 2015, Bobolink abundance steeply declined in grasslands grazed by bison from 2017 to 2019 compared to the 2002–2007 time period in which bison were not present, and the same grasslands were instead grazed by cattle (t = 2.5557, *p* = 0.016; Figure 2). By contrast, during the time period (2002–2019) of our study, Nebraska BBS data showed no significant change in state-wide Bobolink abundance (t = −1.1707, *p* = 0.331). After bison were reintroduced, the annual average number of unique adult Bobolinks in an ~8 ha study site dropped from 12.15 in the 2002–2007 time period to 4.66 from 2017 to 2019, corresponding to a ~62% decline in abundance. The annual average number of unique juvenile Bobolinks dropped from 1.53 in the 2002–2007 time period to 0.25 in the 2017–2019 time period, corresponding to an ~84% decline in productivity. 

### 3.2. Bobolink Abundance in Grazed Grasslands Compared to Hayed Grasslands 

**Figure 3:**
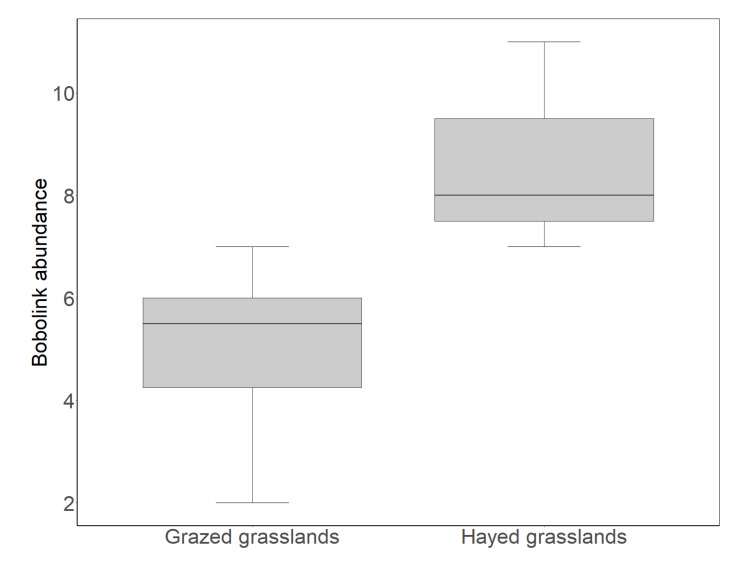
Boxplot showing total Bobolink abundance (unique individuals captured/year) in 2017–2019 on grasslands grazed by bison and cattle compared to grasslands where hay was harvested after the bird breeding season on conservation grasslands in Nebraska, USA. Black lines in each box represent the median value of the abundance, while the lower and upper box edges represent the 25th and 75th percentile of the abundance, respectively. Error bars represent the 5th and 95th percentiles of the abundance.

While we found a significant, empirical decline of Bobolinks over the time period of our study in grasslands where bison reintroduction had taken place, we found no such decline in adjacent grasslands where bison reintroduction did not take place. Over the same time period (2002–2019) that Bobolinks declined in grasslands where bison were reintroduced, Bobolink abundance remained stable in neighboring grasslands where no grazing took place but where hay was harvested after the bird breeding season (t = 1.269, *p* = 0.247). Likewise, Bobolink abundance remained stable in nearby grasslands that were grazed by cattle (t = 1.919, *p* = 0.098). By the conclusion of our study period (2017–2019), Bobolink abundance was higher in hayed grasslands compared to grazed grasslands (t = −3.591, *p* = 0.005; Figure 3). 

### 3.3. Bobolink Abundance and Productivity in Response to Changes in Land Management and Climate Parameters and Their Interactions

**Figure 4:**
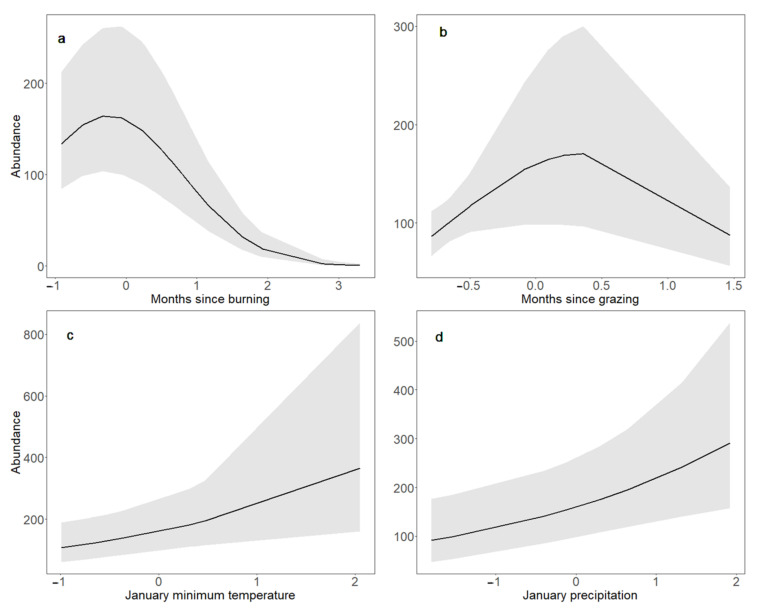
Bobolink adult abundance in response to time since burning (**a**), time since grazing (**b**), January minimum temperature (**c**) and January precipitation (**d**) from 2002 to 2019 in Nebraska, USA. Responses to each predictor were estimated using generalized linear mixed models while keeping the value of other predictors constant at their means (0 for standardized variables). The black line represents the mean, while the shaded area corresponds to the 95% confidence interval.

**Figure 5:**
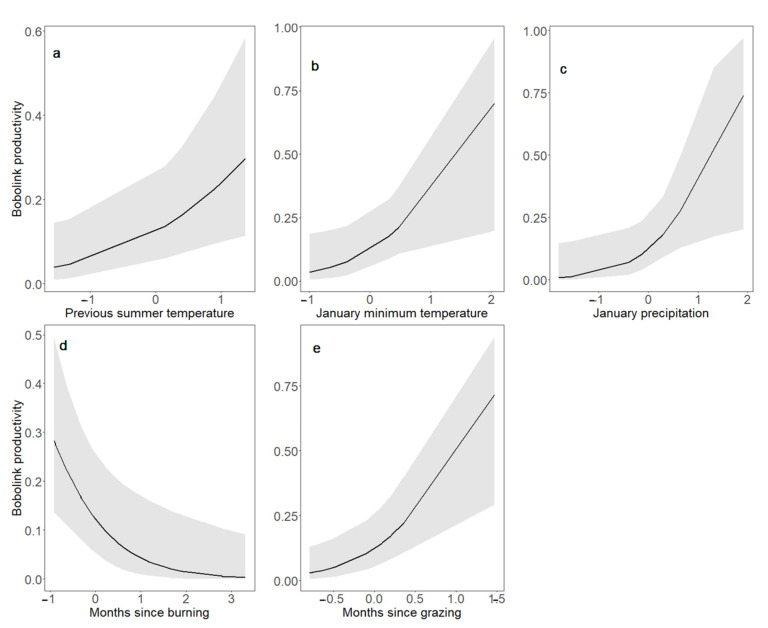
Responses of Bobolink productivity to previous summer temperature (**a**), January temperature (**b**), January precipitation (**c**), months since burning (**d**) and months since grazing (**e**) from 2002 to 2019 in Nebraska, USA. Responses to each predictor were estimated using generalized linear mixed models while keeping the value of other predictors constant at their means (0 for standardized variables). The black line represents the mean, while the shaded area corresponds to the 95% confidence interval.

In addition to Bobolinks’ responses to bison reintroduction, we found that a number of other land management and climate parameters were correlated with changes in Bobolink abundance and productivity (Table 3). In grasslands where patch burning took place, Bobolink abundance peaked immediately after burning and thereafter declined with time since burning (z = −5.451, *p* < 0.001; Figure 4a). In the same manner, Bobolink productivity increased immediately after burning but declined with time since burning (z = 2.684, *p* = 0.007; Figure 5e), paralleling the response of breeding adults. In grasslands that were rotationally grazed, Bobolink abundance peaked after the departure of cattle and then declined with time since grazing (z = −2.328, *p* = 0.020; Figure 4b). In addition, Bobolink abundance increased with both increasing minimum January temperature (z = 2.509, *p* = 0.012; Figure 4c) and increasing January precipitation (z = 2.760, *p* = 0.006; Figure 4d). Mirroring Bobolink abundance, Bobolink productivity likewise increased with increasing minimum January temperature (z = 2.202, *p* = 0.028; Figure 5b) and increasing January precipitation (z = 2.131, *p* = 0.033; Figure 5c).

Bobolink productivity was negligible in the presence of active grazing and increased over time after the departure of cattle (z = −2.404, *p* = 0.016; Figure 5d). Bobolink productivity also increased with increasing average previous breeding season temperature (z = 2.162; *p* = 0.031; Figure 5a). In addition, we found that Bobolink productivity was positively correlated with the interaction of August PDSI (which represents cumulative drought conditions during the summer breeding season [May–July]), with both time since active grazing (z = 2.517, *p* = 0.012) and grazing intensity (z = 2.768, *p* = 0.006). This indicates that wetter summer conditions and past grazing intensity enhanced the increases in productivity exhibited by Bobolinks following the departure of grazers from their breeding grounds. 

## 4. Discussion

Contrary to our hypothesis that reintroducing bison would improve the conservation value of grassland as bird habitat and contribute to increased numbers of Bobolinks, we found that four years after bison reintroduction, Bobolink abundance had declined by 62% and productivity had declined by 84%. Over the same time period, state-wide Bobolink abundance did not decline, but remained stable. Bobolink abundance also remained stable in adjacent grasslands where hay was harvested after the bird breeding season, as well as in nearby grasslands grazed by cattle. Taken together, these data suggest that the severe Bobolink declines we observed in our study area were driven by bison reintroduction.

Most breeding Bobolinks do not appear to be able to persist or successfully nest in bison-grazed grasslands with densities of bison comparable to those in our study area (up to ~1 bison/3 ha). By contrast, the U.S. National Park Service maintains bison at a much lower population density in Yellowstone National Park (~1 bison/200 ha) and plans to introduce bison to South Dakota’s Rosebud Sioux Reservation with a density of up to ~1 bison/12 ha [6]. Such lower bison densities likely have a higher probability of being compatible with the conservation of Bobolinks and birds with similar life histories [45]. However, maintaining lower bison densities for breeding herds in the absence of wild predators [e.g., wolves] requires intensive management, including regular, systematic removal of bison [34,35]. While the costs and logistics associated with such management may be borne by government agencies with sufficient resources, they may be impractical or beyond the capacities of many smaller, private conservation grasslands with budget and staff limitations.

Bobolinks have indicated a propensity for site fidelity in other studies that was corroborated by our recapture rate for Bobolinks, which was comparable to the general recapture rate for Bobolinks in the entire MAPS program [37,38]. The average proportion of juvenile birds (17%) we found was slightly higher than the proportion of juvenile Bobolinks (7–11%) found in the entire MAPS program, but likewise indicative of the low productivity that may be an important driver of Bobolink population declines [37]. Whereas Bobolink clutch sizes typically include ~5 eggs, the number of fledglings that survive to be independent juveniles (our basis for estimating productivity) may be much lower (e.g., 2.6/nest in New York and 2.7/nest in Wisconsin grasslands; [34,38]). Our overall average finding of ~2 independent juveniles/female Bobolink is comparable, but lower, and may be partly related to the male-biased sex ratio we found of ~2 males to 1 female breeding Bobolink.

### 4.1. Bobolink Abundance and Productivity Exhibited Steep Declines after Bison Reintroduction

Four years after bison were reintroduced in our study area, Bobolink abundance declined by nearly two-thirds compared to Bobolinks’ earlier abundance on the same grasslands when they were grazed by cattle (Figure 2). Previous studies have found that Bobolink declines intensify in response to increased grazing intensities [27,92], suggesting that overgrazing and associated negative impacts contributed to the Bobolink declines we found after bison reintroduction. Overgrazing negatively affects breeding birds not only by trampling but also by decreasing protective cover for ground-nesting birds such as Bobolinks, leaving their nests more visible to nest predators [93], which may facilitate increased rates of nest predation [94,95,96] and/or parasitism by Brown-headed Cowbirds (*Molothrus ater*) [97]. In addition to eliminating potential nesting and roosting locations for grassland birds [27,38], overgrazing may also cause declines in plant communities and arthropods, reducing food resources for Bobolinks and other grassland birds [44,98]. 

After bison reintroduction, Bobolinks’ productivity declined even more steeply than their abundance, falling by more than four-fifths. The presence of active grazing in grasslands may decrease birds’ nest success, in part because unlike adult birds, nests and chicks cannot move to avoid being trampled or depredated, and many grassland bird species appear to avoid nesting in grasslands with active grazing [97]. Because Bobolinks nest on the ground, the presence of active grazing and its impacts directly affect Bobolinks’ potential for successfully nesting and raising juvenile birds until they develop flight skills and independence [27,42]. In rotational grazing systems in Vermont, for example, predation and trampling by cattle accounted for a significant proportion (32%) of nest failures, significantly lowering Bobolink productivity [99]. While light bison grazing may contribute to more complex plant communities and higher habitat structure variability, potentially benefiting Bobolinks [27,45], heavy grazing drives departures of breeding adults and directly contributes to nest loss [44,98], as appears to have taken place in our study. 

### 4.2. Bobolink Abundance and Productivity Were Lower in Grazed Than Hayed Grasslands, but Increased after the Departure of Grazers 

The significant decline of Bobolinks we observed over the time period of this study occurred specifically where bison reintroduction had taken place (Figure 2), and not in adjacent grasslands without bison. Bobolink abundance remained stable in adjacent grasslands where hay was harvested after the bird breeding season, as well as in grasslands grazed by cattle. By the conclusion of our study period, hayed grasslands supported significantly higher numbers of Bobolinks than grazed grasslands (Figure 3). In grazed grasslands, Bobolink adult abundance peaked after the departure of grazers (Figure 4), a response consistent with other research showing that Bobolinks appear to benefit from post-grazing responses of grasslands, such as increases in plant growth and arthropod activity [27,100,101,102,103,104]. While recent grazing appears to benefit Bobolinks, active grazing is detrimental, and grazers must first depart a grassland for this response to occur. 

Bobolink productivity was negligible in the presence of active grazing, but increased over time following the departure of cattle (Figure 5). This pattern demonstrates the importance not only of managing grasslands to avoid overgrazing but the crucial importance of avoiding grazing disturbance to breeding birds to allow nest success and subsequent recruitment of juvenile birds into the breeding population. In extended or continuous grazing systems, Bobolink productivity appears to be severely impaired, likely due to potential trampling, and increased vulnerability of bird and nests to predators and parasites. Previous studies in the Great Plains found that Bobolinks responded positively to moderate grazing in tallgrass prairie, such as our study area, and increased in response to short-duration (e.g., periods of several months) grazing [27,92,105]. 

~150 years ago, bison were the keystone native grazers in these grasslands, and their presence was ephemeral, as part of migratory herds [7,11]. In contrast to the free-roaming behavior historically present in bison, modern management systems may lead to the containment of bison at high densities in limited areas, such as in our study area, for extended periods, or year-round. Whereas cattle grazing in grasslands in our study area was by cow-calf pairs that grazed for short durations before departing, grazing bison remained on grasslands for extended periods, including though the winter, and were actively breeding, which resulted in the bison herd doubling in size over a four-year period. 

Our data show that where Bobolinks had successfully nested and raised young in grasslands grazed rotationally by cattle, they declined rapidly and steeply after cattle were replaced by bison. Although periodic disturbances such as grazing, hay harvesting, and patch burning maintain habitat quality for grassland birds (Figure 4 and Figure 5), Bobolinks and birds with similar life histories decline with increasing grazing intensity on their breeding grounds, particularly when grazers are present during the bird breeding season [27,106,107,108]. While bison reintroduction appeared to drive Bobolink declines under these conditions, reducing the number of bison would likely mitigate their negative impacts on Bobolinks. Moreover, arranging for the departure of grazers, whether bison or cattle, prior to the bird breeding season would reduce disturbances to breeding birds and reduce the risks of nests and chicks exposed to trampling and predation due to overgrazing and its associated impacts on birds and bird habitat.

Managing grasslands to avoid overgrazing in general and disturbance to nesting and juvenile birds in particular, whether from grazing by bison or cattle, hay harvesting, or patch burning, makes a critical difference for Bobolinks and other ground-nesting birds [22,40]. Our findings show that both Bobolink abundance and productivity significantly increased following the departure of grazing animals (Figure 4 and Figure 5), while both Bobolink abundance and productivity exhibited significant declines when cattle grazing was replaced with bison grazing in our study area (Figure 2). Thus, overgrazing and related impacts following bison reintroduction apparently undermined the ability of Bobolinks to persist, nest, and raise young on grasslands where they were formerly abundant.

### 4.3. Bobolink Abundance and Productivity Increased following Warmer, Wetter Winters, and after Patch Burning; Bobolink Productivity Also Increased in Response to Warmer, Wetter Summers

In addition to the effects of bison reintroduction, we found that other land management and climate predictors also influenced Bobolink populations in our study area. Both Bobolink abundance and productivity increased immediately after patch burning but decreased over time thereafter (Figure 4 and Figure 5), corroborating other research showing that patch burning may provide temporary benefits to Bobolinks [27]. Recent burning may help deter predators [104] by reducing suitable habitat for nest predators that prefer thick or woody vegetation [105]. While periodic patch burning may thus be used to successfully maintain Bobolink habitat [41,109], bison prefer to graze in burned areas, which may magnify their effects [10] and contribute to their negative effects on Bobolinks in this study. 

During the time period of our study (2002–2019), precipitation significantly increased in our study area, consistent with climate change predictions [64], and there was a non-significant trend towards warmer temperatures. Bobolink population dynamics exhibited significant responses to local climatic changes in our study area, with both abundance and productivity increasing with increasing January minimum temperatures and precipitation (Figure 4 and Figure 5). January was both the coldest and driest month of the year in our study area, and during the time period of this study, minimum January temperature always fell below −4 °C [80]. Colder Januarys may indirectly affect vegetation production via soil moisture, if temperatures are sufficiently low that the soil remains frozen for most of the winter, and soil water levels do not recharge until spring. Conversely, warmer temperatures and increased precipitation directly contribute to increases in arthropods [42,89], plants, and seeds [101,102,110] that provide a large proportion of Bobolinks’ food. Vegetation type, height, heterogeneity and density all play direct roles in availability of nest locations for birds [89], level of nest cover [44], concealment from predators ([110]; but see [111]), availability of singing perches for males [110], and in mitigating the impacts of extreme weather events or periods of resource scarcity [112,113].

Bobolink productivity demonstrated positive responses to warmer and wetter summer conditions. Specifically, Bobolink productivity increased with warmer temperatures during the previous breeding season (Figure 5), highlighting the carry-over effects of weather on Bobolinks in addition to the impacts of weather on grasslands and birds in real time [63,64,89]. Further, Bobolink productivity showed a positive correlation with the interaction of August PDSI, which represents cumulative drought conditions during the summer breeding season (May–July), and both time since active grazing and grazing intensity. At the highest values of grazing intensity (1.05 AUM/ha) and in the least-recently grazed sites, wetter breeding season conditions were positively correlated with Bobolink productivity. During drier breeding season conditions, Bobolink productivity may be limited by decreased food availability, as dry conditions decrease plant biomass and aboveground primary productivity [114,115], leading to reductions in arthropods that constitute a significant source of prey items provisioned to Bobolink nestlings [29,42,116]. Under such conditions, food scarcity may lead to direct nestling mortality through starvation, slowed nestling growth rates [89], parental abandonment of nests [114], and nestlings begging more conspicuously, thus attracting the attention of nest predators [29]. 

By contrast, where warmer winter temperatures and higher levels of precipitation allow plants to start growing, flowering, and producing seeds earlier in the year, Bobolinks arriving from spring migration to breed may benefit from improved conditions for nesting and raising chicks that in turn results in greater Bobolink productivity. In addition, our findings that warmer summers are followed by increased Bobolink productivity in subsequent years, and that Bobolink productivity in heavily grazed grasslands increased in response to wetter summer conditions, suggest that the regional trend toward warmer and wetter winters and summers in this region [63,64] has the potential to benefit both Bobolink abundance and productivity. 

Given the significant positive responses Bobolink populations in our study area exhibited to wetter, warmer winters and summers, we might have expected corresponding increases in Bobolink abundance and productivity in response to the significantly higher levels of precipitation and trend towards warmer temperatures that transpired over the time period of our study. The fact that we found no such empirical increases in Bobolinks in hayed or cattle-grazed grasslands may be because the strength of this interaction was insufficient to benefit Bobolinks over the time period of this study, but warrants future investigation. However, in grasslands where bison were reintroduced, the negative consequences for Bobolinks would likely overshadow any potential benefits to Bobolinks from regional climate change.

## 5. Conclusions and Recommendations

Our finding that Bobolinks declined following bison reintroduction on private conservation grasslands adds to previous research that identified declines in other grassland bird species and population parameters following bison reintroductions [10,36]. While bison reintroduction may benefit some bird species [10,117] and offers people a sense of connection with conservation [7,10], grasslands with high bison densities are not compatible with the objective of managing grasslands for bird species of conservation concern such as Bobolinks. As breeding bison populations may grow quickly, grasslands grazed by bison may be prone to overgrazing, trampling, and other negative impacts on birds, rendering grasslands unsuitable for Bobolinks and other species with similar life histories. As identifying and managing for conditions to improve Bobolink productivity are a conservation priority [37], we recommend that conservation land managers avoid overgrazing grasslands and prioritize protecting birds from disturbance during the nesting season, including by grazing bison or cattle, haying, and burning [27,118,119,120], to maximize breeding bird abundance and productivity. Favorable grassland management practices for Bobolinks and birds with similar life histories appear to include light, short-duration rotational grazing as well as hay harvesting and patch burning outside the bird breeding season.

Bobolinks and other migratory birds are exhibiting responses to regional climate change, which has resulted in a trend towards warmer temperatures and significantly higher precipitation in our study area and region [63,64,120,121]. Despite Bobolink abundance and productivity exhibiting positive correlations with wetter and warmer conditions, however, Bobolink populations did not exhibit empirical increases over the time period of our study. Moreover, the severe declines of Bobolinks following bison reintroduction in our study area suggests that the negative effects of bison on Bobolinks likely outweigh any potential benefits to Bobolinks in this area from regional climate change. Ideally, future studies will investigate both Bobolink and other grassland bird species’ responses to lower densities of bison over time to determine bison densities that might be compatible with the conservation of breeding Bobolinks and other grassland birds of conservation concern in this and other regions, habitats, and management contexts. Replicated studies, including nest monitoring, which consider climatic variation, predators, and parasites would provide insights into mechanisms driving bird population change and how these may be addressed through adaptive management for bird conservation.

## Figures and Tables

**Table 1:** Land management and habitat parameters tested as predictors of change in Bobolink abundance and/or productivity between 2002 and 2019 on conservation grasslands in Nebraska, USA.

Variable	Type	Definition
Months since grazing	Continuous	Months since the site was last grazed
Months since haying	Continuous	Months since the site was last hayed
Months since burning	Continuous	Months since the site was last burned
Grazing intensity	Continuous	Grazing effort (AUM/ha) of the site at the time the sample was taken
Grassland type	Categorical	Remnant: grasslands that were never tilled for agriculture; restored: grasslands replanted after being previously used for agriculture
Tree coverage	Continuous	Percent tree cover within a 200 m radius of a site

**Table 2:** Climate parameters tested as predictors of change in Bobolink abundance and/or productivity between 2002 and 2019 on conservation grasslands in Nebraska, USA.

Variable	Type	Definition
July maximum temperature	Continuous	Average daily maximum temperature (°C) in July
January minimum temperature	Continuous	Average daily minimum temperature (°C) in January
May precipitation	Continuous	Total precipitation (mm) in May
January precipitation	Continuous	Total precipitation (mm) in January
August PDSI	Categorical	Breeding season (May–July) drought intensity
Previous August PDSI	Categorical	Previous breeding season (May–July) drought intensity
August–April PDSI	Categorical	Nonbreeding season (August–April) drought intensity
Previous breeding season temperature	Continuous	Average daily temperature (°C) in the previous breeding season

**Table 3:** Bobolink adult and juvenile AIC model results from generalized linear mixed models testing land management and/or climate predictors of changes in Bobolink abundance and productivity.

Model Type	Formula	Family	ΔAIC
Bobolink adult abundance	Adult abundance ~ Period + Grazing intensity + Months since grazing + Months since grazing^2^ + Months since haying + Months since burning + Months since burning^2^ + Percent trees + January minimum temperature + January precipitation + July maximum temperature + May precipitation + August PDSI + August-April PDSI + (1|site)	Negative binomial corrected for overdispersion	0.0
	Adult abundance ~ Period + Grazing intensity + Grazing intensity^2^ + Months since grazing + Months since grazing^2^ + Months since haying + Months since haying^2^ + Months since burning + Months since burning^2^ + Percent trees + January minimum temperature + January precipitation + July maximum temperature + July maximum temperature^2^ + May precipitation + August PDSI + August PDSI^2^ + August-April PDSI + (1|site)	Negative binomial corrected for overdispersion	3.0
	Adult abundance ~ Period + Grazing intensity + Months since grazing + Months since haying + Months since burning+ Percent trees + January minimum temperature + January precipitation + July maximum temperature + May precipitation +August PDSI + August-April PDSI + (1|site)	Negative binomial corrected for overdispersion	31.6
	Adult abundance ~ Period + Grazing intensity + Months since grazing + Months since haying + Months since burning+ Percent trees + January minimum temperature + January precipitation + July maximum temperature + May precipitation +August PDSI + August-April PDSI + (1|site)	Quasipoisson	4367.9
Bobolink productivity	Bobolink productivity ~ Period + landuse + Percent trees + Previous breeding season temperature + January minimum temperature + January precipitation + July maximum temperature + Months since haying x August PDSI + Months since burning x Months since grazing + August PDSI x Grazing intensity + August PDSI x Months since grazing + (1|site)	Binomial	0
	Bobolink productivity ~ Period + landuse + Grazing intensity + Months since grazing + Months since haying + Months since burning + Percent trees + Previous breeding season temperature + January minimum temperature + January precipitation + July maximum temperature + August–April PDSI + August PDSI + (1|site)	Binomial	11.3
	Bobolink productivity ~ Period + landuse + Grazing intensity + Grazing intensity^2^ + Months since grazing + Months since grazing^2^ + Months since haying + Months since haying^2^ + Months since burning + Months since burning^2^ + Percent trees + Percent trees^2^ + January minimum temperature + January precipitation + July maximum temperature + July maximum temperature^2^ + August–April PDSI + August PDSI + August PDSI^2^ + (1|site)	Binomial	21.4

## Data Availability

Data supporting the results can be obtained from the authors, including Bobolink, land management, and climate data presented in https://www.mdpi.com/article/10.3390/ani11092661/s1, Table S1. Additional climate data may be obtained from NOAA’s “Climate at a Glance” database: https://www.ncdc.noaa.gov/cag/ (accessed on 10 August 2021).

## Supplementary Materials

The following are available online at https://www.mdpi.com/article/10.3390/ani11092661/s1, Table S1: Bobolink data from 2002–2019 (abundance [numbers of unique adult birds] and productivity [numbers of unique juvenile birds]; Crane Trust land management data from 2002–2019 (bison grazing intensity and duration, cattle grazing intensity and duration, hay harvest timing, and patch burning timing); NOAA climate data for station KGRI [south-central Nebraska] from 2002–2019 (temperature, precipitation, Palmer Drought Severity Index [PDSI]).
